# Magnetic and Magneto-Caloric Properties of the Amorphous Fe_92−x_Zr_8_B_x_ Ribbons

**DOI:** 10.3390/ma13235334

**Published:** 2020-11-25

**Authors:** Xin Wang, Qiang Wang, Ben Zhen Tang, Ding Ding, Li Cui, Lei Xia

**Affiliations:** 1 College of Engineering, Shanghai Polytechnic University, Shanghai 201209, China; wangxin2020@126.com (X.W.); cuili@sspu.edu.cn (L.C.); 2 Institute of Materials, Shanghai University, Shanghai 200072, China; mat_wq@shu.edu.cn (Q.W.); amtbpeace@yeah.net (B.Z.T.); xialei@shu.edu.cn (L.X.); 3 Center for Advanced Microanalysis, Shanghai University, Shanghai 200444, China

**Keywords:** metallic glasses, magnetocaloric effect, Curie temperature, the adiabatic temperature rise

## Abstract

Magnetic and magnetocaloric properties of the amorphous Fe_92−x_Zr_8_B_x_ ribbons were studied in this work. Fully amorphous Fe_89_Zr_8_B_3_, Fe_88_Zr_8_B_4_, and Fe_87_Zr_8_B_5_ ribbons were fabricated. The Curie temperature (*T_c_*), saturation magnetization (*M_s_*), and the maximum entropy change with the variation of a magnetic field (−Δ*S_m_^peak^*) of the glassy ribbons were significantly improved by the boron addition. The mechanism for the enhanced *T_c_* and −Δ*S_m_^peak^* by boron addition was studied.

## 1. Introduction

With the rising concerns on environmental pollution and the higher and higher cost of energy, it is an urgent need recently to develop energy-saving and environmental-friendly materials, such as new energy storage materials, magnetocaloric materials, giant impedance materials, thermoelectric materials, and magneto-strictive materials [1,2,3,4,5]. Magnetocaloric materials are the materials that exhibit an adiabatic temperature change when they experience a magnetization or demagnetization process, which is called the magnetocaloric effect (MCE). Magnetic refrigerators using the magnetocaloric alloys or compounds as working materials are believed to be more compact (because of solid refrigerant) and efficient (due to their lower energy consumption) than the traditional vapor compression/expansion refrigerator. Furthermore, the magnetic refrigerators are considered to be safer to the environment because they do not emit ozone-depleting gases [6,7]. Therefore, the magnetocaloric materials have recently attracted more and more attention, and as a result, a great number of magnetocaloric alloys or compounds have been developed in the past several decades [8,9,10].

The amorphous alloys, which exhibit higher corrosion resistance and better mechanical properties than the crystalline alloys, are considered to be suitable candidates for magnetic refrigerants because they exhibit rather broad magnetic entropy change (−Δ*S_m_*) peak [11,12]. The broadened −Δ*S_m_* peak and the resulted high value of refrigeration capacity (*RC*) lead to a rather wide working temperature range and a large amount of cooling, which is an important indicator that helps to obtain the maximum cooling capacity in the Ericsson cycle [13]. The amorphous alloys can be fabricated within a large compositional range, indicating that the Curie temperature (*T_c_*) and properties, depending on the alloy compositions, can be easily tuned [14].

Among the amorphous alloys that have been studied so far, Gd-based amorphous alloys and some high-entropy alloys show good reversible magnetocaloric effects [15,16,17]. Usually, the large maximum −Δ*S_m_* (−Δ*S_m_^peak^*) value of these amorphous alloys only obtained at low temperatures and their high cost limit their industrial applications. Although some amorphous alloys with reduced Gd content can achieve application conditions in the room temperature range, they still have shortcomings in terms of cost, magnetocaloric effect, and forming ability [18,19]. In contrast, the low-cost transition metal (TM)-based amorphous alloys represented by the Fe-based amorphous alloy gained more and more attention.

In previous studies, Fe_100−x_B_x_ (x = 12–28) amorphous alloy exhibits good soft magnetic properties [20,21], However, almost all the compositions of the Curie temperatures, *T_c_*, are above room temperature. By adding Nb, Y, Nd, or Mn elements to amorphous system, the Curie temperature is greatly reduced, but it fails to increase the magnetic entropy change value of the alloy or even deteriorate [22]. On the other hand, FeZr binary system alloys exhibit excellent soft magnetic properties near room temperature and are accompanied with a −Δ*S_m_^peak^* close to about half of the Gd [23,24]. As a result, substitution of Fe by transition metals Nb, Mn, Y, or metalloid elements B can significantly change the magnetic properties [25,26,27]. In particular, the addition of B can even make the alloy appear ferromagnetic near room temperature. For the purpose of meeting the requirements of magnetic refrigerants in a domestic refrigerator, recently, many multicomponent Fe-based amorphous alloys with excellent magnetocaloric properties have been synthesized based on the ternary Fe-Zr-B glass-forming alloys [27,28,29,30]. The lower Zr content Fe-B-Zr amorphous alloys usually have a lower MCE with the −Δ*S*_m_*^peak^* about 1.04 J K^−1^ kg^−1^ for a field change of 0–15 kOe in the Fe_94−x_Zr_6_B_x_ (x = 5, 6, 8, and 10) amorphous alloys. When the Zr content is increased, the Δ*S*_m_ of the alloy becomes significantly improved: for instance, the magnetic properties, phase transitions, and MCE were systematically studied in amorphous Fe_89−x_B_x_Zr_11_ (x = 0–10) alloys and the *T_c_* got enhanced with B addition and the −Δ*S_m_* value to be about 1.73 J K^−1^ kg^−1^ for the Fe_79_Zr_11_B_10_ sample. Meanwhile, the addition of a series of 3D elements, such as Cu, Cr, Mn, Co, Ni..., also obtained a series of FeZrB-based amorphous alloys with a good Δ*S_m_* value near room temperature [31,32,33,34,35,36]. Recently, related studies have shown that FeZrB(Cu,Co...) amorphous alloys also have dispersed nanocrystalline particles on the amorphous matrix [37,38]. Appropriate selection and control methods of heat treatment can make nanocrystalline particles aggregate and grow, further, by selectively removing the surface nanocrystalline particles, the amorphous materials with nanoporosity on the surface can be obtained. However, after the selective dealloying treatment of nanocrystalline amorphous alloys, it is found that the magnetization of the alloys is improved. This is mainly related to the increase in the concentration of ferromagnetic atoms in the system, which also provides a way to further improve the magnetic properties of the amorphous alloys.

Through the previous study on the Fe_88_Zr_8_B_4_ amorphous alloy [30,39,40], it is found that a moderate Zr content makes the alloy exhibit good magnetic properties near room temperature. However, the mechanism for their good magnetocaloric properties has not been investigated systematically. Therefore, the detailed investigation on the magnetic and magnetocaloric properties of Fe-Zr-B ternary metallic glasses may be helpful for the understanding of the tailorable magnetic and magnetocaloric properties near room temperature in the multicomponent Fe-Zr-B-based metallic glasses. In the present work, we fabricated Fe_92−x_Zr_8_B_x_ (x = 3, 4, 5) amorphous samples in the shape of ribbons with an average thickness of 0.04 mm. Magnetic properties of the amorphous samples were measured and their magnetocaloric properties were obtained. The dependence of *T_c_* as well as −Δ*S_m_^peak^* on the composition of the metallic glasses were constructed for the purpose of revealing the mechanism involved.

## 2. Experiments

Alloy ingots with a nominal composition of Fe_92−x_Zr_8_B_x_ (x = 3, 4, 5) were prepared by arc melting a mixture of high-purity (99.95 wt%) Fe, Zr metallic pieces and Fe-B pre-alloy for at least four times in a non-consumable electrode high vacuum arc melting furnace. Fe_92−x_Zr_8_B_x_ ribbons with an average thickness of ~0.04 mm were prepared by ejecting the melts from the quartz tube to the surface of a rotating copper wheel under a pure Ar atmosphere. The surface speed of the copper wheel was optimized at 30 m/s. Structure of the ribbons was checked by X-ray diffraction (XRD) using the *K_α_* radiation of Cu on a Rigaku diffractometer (model D/max-2550) (Rigaku, Tokyo, Japan). Thermal properties about the glass transition temperature (*T_g_*), crystallization temperature (*T_x_*) and the liquidus temperature (*T_l_*) of the amorphous ribbons were measured by a Netzsch DSC-404C differential scanning calorimetry (DSC) (Netzsch, Selb, Germany) under a purified argon atmosphere at a heating rate of 20 K/min. Microstructures of the amorphous ribbons were observed by a JEOL JEM-2010F (JEOL, Tokyo, Japan) high-resolution electron microscope (HREM). The specimens for HREM observations were prepared by ion-polishing under a pure argon atmosphere using the GATAN 691 precision ion-polishing system (AMETEK, Berwyn, PA, USA). Magnetic properties of the as-spun ribbons were measured by a Quantum Design Physical Properties Measurement System (Ever cool II): the temperature dependence of the magnetization (M-T) curves were obtained under a field of 0.03 T in the cooling process; hysteresis loops were measured under a field of 5 T at 10 K and 380 K, respectively; isothermal magnetization (M-H) curves were obtained at various temperatures under a field of 5 T. The heat capacity (*C_p_*(*T*)) of the Fe_87_Zr_8_B_5_ amorphous ribbon was also measured by PPMS near its *T_c_* under a zero magnetic field.

## 3. Results and Discussion

**Figure 1:**
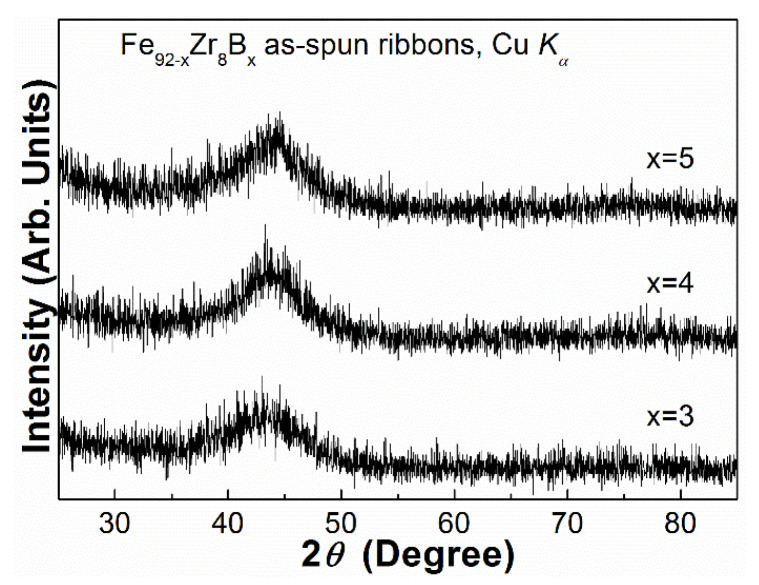
XRD patterns of the Fe_92−x_Zr_8_B_x_ (x = 3, 4, 5) as-spun ribbons.

X-ray diffraction patterns of the Fe_92−x_Zr_8_B_x_ (x = 3, 4, 5) ribbons are presented in Figure 1. Only one typical broadened diffraction hump was observed between 2*θ* of 30° and 35° on each pattern; and the absence of visible crystalline peaks are present on the XRD curves of the ribbons. It indicates that the Fe_92−x_Zr_8_B_x_ ribbons are fully amorphous structures.

**Figure 2:**
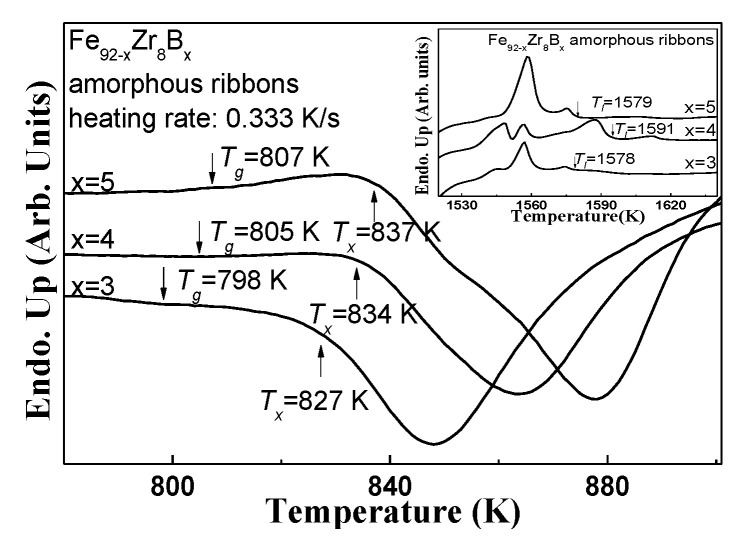
Differential scanning calorimetry (DSC) traces of the Fe_92−x_Zr_8_B_x_ (x = 3, 4, 5) alloys with the heating curves obtained at 20 K/min; the inset is the melt DSC trace.

The amorphous feature of the Fe_92−x_Zr_8_B_x_ ribbons prepared under the linear velocity of 30 m/s can be further confirmed from their differential scanning calorimetry (DSC) trace (Figure 2). The obvious endothermic glass transition behaviors before the crystallization and the visible crystallization exothermic peak (see the small figure in Figure 2) also verify the amorphous characteristics of the ribbon. As seen from the DSC trace, the onset temperatures of glass transition (*T_g_*) of Fe_92−x_Zr_8_B_x_ (x = 3, 4, 5) are about 798 K, 805 K, 807 K; and crystallization (*T_x_*) is about 827 K, 834 K, and 837 K, respectively.

**Figure 3:**
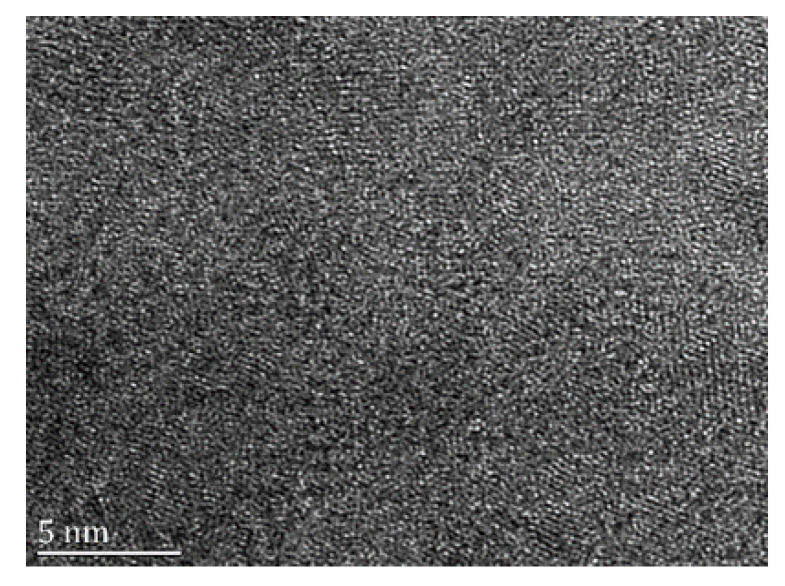
High-resolution electron microscope (HREM) micrograph of Fe_88_Zr_8_B_4_ sample.

In order to verify the above assumption more intuitively, high-resolution electron microscope (HREM) micrographs of the Fe_88_Zr_8_B_4_ sample were performed and depicted in Figure 3. The HREM image reveals the fully amorphous characteristics with only short-range order in the disordered matrix. Similar features in XRD patterns and the DSC curves of these samples indicates the approximate structural features in all other samples studied.

**Figure 4:**
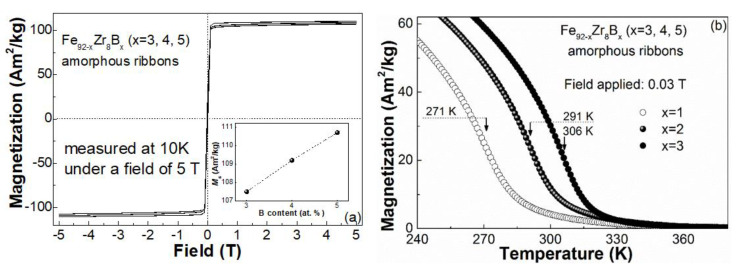
(**a**) Hysteresis loops of the Fe_92−x_Zr_8_B_x_ (x = 3, 4, 5) amorphous ribbons measured at 10 K under a field of 5 T; the inset is the relationship between the *M_s_* and x. (**b**) The *M*-*T* curves of the Fe_92−x_Zr_8_B_x_ (x = 3, 4, 5) amorphous alloys measured under a field of 0.03 T.

Coercivity and saturation magnetization (*M_s_*) of the Fe_92−x_Zr_8_B_x_ (x = 3, 4, 5) glassy ribbons were obtained from the hysteresis loops measured at 10 K under 5 T. As shown in Figure 4a, the nearly zero coercivity of all the Fe_92−x_Zr_8_B_x_ (x = 3, 4, 5) glassy samples indicates that the metallic glasses are soft magnetic properties at 10 K. *M_s_* of the Fe_92−x_Zr_8_B_x_ (x = 3, 4, 5) glassy ribbons obtained from their hysteresis loops are about 107.5 Am^2^/kg for x = 3, 109.2 Am^2^/kg for x = 4, and 110.7 Am^2^/kg for x = 5, respectively. The dependence of *M_s_* on the boron content of the three glassy samples, as plotted in the inset of Figure 4a, shows a roughly linear relationship between *M_s_* and x. The increasing *M_s_* with x in the Fe_92−x_Zr_8_B_x_ metallic glasses is most likely related to the improved Fe-B interactions with increasing B content [27,39]. The enhanced *M_s_* implies the improvement of MCE by boron addition in the Fe_92−x_Zr_8_B_x_ amorphous alloys because *M_s_* or −Δ*S_m_* depends on the ordering of magnetic moments in metallic glasses upon magnetization.

In addition, unlike the rare earth (RE)-transition metal (TM)-based (RE-TM-based) metallic glasses, the enhanced interaction by boron addition may result in the improvement of *T_c_* in Fe_92−x_Zr_8_B_x_ glassy alloys because *T_c_* of the Fe-based metallic glass samples primarily depend on the 3*d*-3*d* direct interaction [27,29,34,35,36,37]. Figure 4b shows the variation of magnetization on the temperature (*M*-*T* curves) of the Fe_92−x_Zr_8_B_x_ glassy samples measured under 0.03 T. *T_c_* derived from the *M*-*T* curves is about 271 K for Fe_89_Zr_8_B_3_, 291 K for Fe_88_Zr_8_B_4_, and 306 K for Fe_87_Zr_8_B_5_ amorphous ribbons. The Curie temperatures of the glassy samples were located in the working temperature range of a domestic refrigerator, which indicates that the Fe_92−x_Zr_8_B_x_ glasses ribbons may be the good working medium of magnetic refrigeration when the −Δ*S_m_^peak^* of these alloys are high enough.

**Figure 5:**
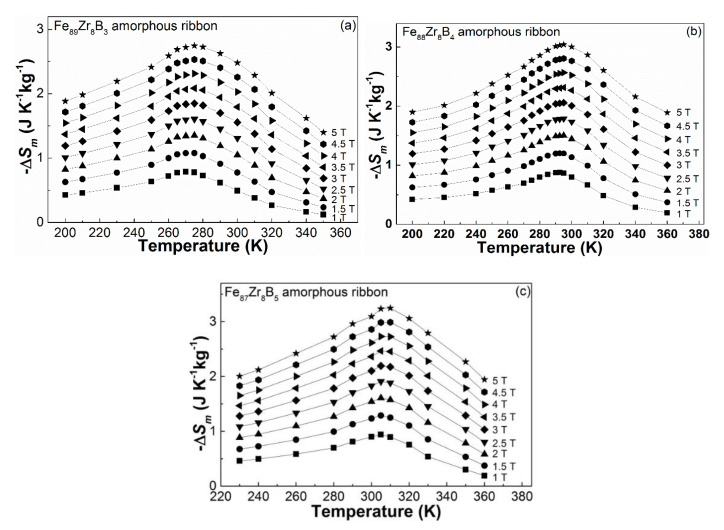
−Δ*S_m_*-*T* curves of the Fe_92−x_Zr_8_B_x_ (x = 3, 4, 5) amorphous ribbons under various magnetic fields: (**a**) x = 3, (**b**) x = 4, and (**c**) x = 5.

**Figure 6:**
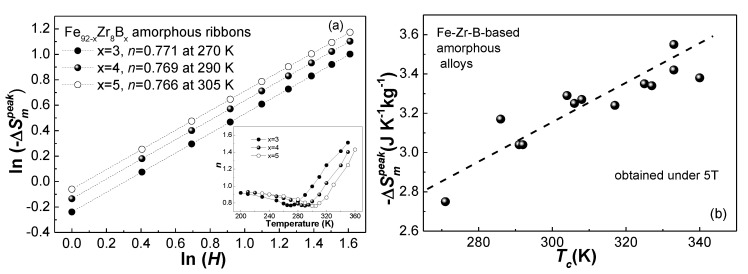
(**a**) The −Δ*S_m_^peak^*∝*H^n^* relationship of the Fe_91−x_Zr_9_B_x_ (x = 3, 4, 5) amorphous alloys at *T_c_*, the inset is the *n*-*T* curves of the three amorphous alloys; (**b**) the −Δ*S_m_^peak^* (under 5 T) vs. *T_c_* plots and the linear fitting (dash line) these plots in several Fe-Zr-B-based amorphous samples (listed in Table 1).

By measuring the isothermal magnetization (*M*-*H*) curves of the Fe_92−x_Zr_8_B_x_ glassy ribbons at various temperatures, we can calculate the −Δ*S_m_* of these amorphous alloys. Figure 5 shows the −Δ*S_m_* plots at different temperatures under the magnetic fields of 1 T, 1.5 T, 2 T, 2.5 T, 3 T, 3.5 T, 4 T, 4.5 T, and 5 T. According to the trend, with a flat and continuously changing value, shown in the −Δ*S_m_*-*T* curves, it can be seen that the Fe_92−x_Zr_8_B_x_ (x = 3, 4, 5) metallic glasses exhibit the secondary magnetic phase transition features of a soft magnetic alloy. −Δ*S_m_^peak^* values of the glassy ribbons under 1 T, 2 T, 3 T, 4 T, and 5 T are listed in Table 1. The −Δ*S_m_*∝*H^n^* relationship for the three samples were constructed and the *n* values at different temperatures were obtained. Figure 6a illustrates the ln(−Δ*S_m_*) vs. ln(*H*) plots near the Curie temperature of the three glassy samples and their linearly fitted lines. *n* is about 0.771 for Fe_89_Zr_8_B_3_ at 270 K, 0.769 for Fe_88_Zr_8_B_4_ at 290 K, and 0.766 for Fe_87_Zr_8_B_5_ at 305 K. The values of *n* near *T_c_* are approximately consistent in the alloys, and the alloys with fully amorphous structures exhibit 2rd magnetic phase transition [17,41,42]. The *n*-*T* curves for the Fe_92−x_Zr_8_B_x_ glassy samples, seen in the inset of Figure 6a, display typical magnetocaloric behaviors of soft magnetic metallic glasses: *n* is nearly 1 at low temperature when the sample is ferromagnetic, then gradually reduces to a minimum value near *T_c_*, and finally increases dramatically to a value up to 2 at the paramagnetic state [18,43].

−Δ*S_m_^peak^* of Fe_92−x_Zr_8_B_x_ amorphous alloys, as predicted above, increases with boron addition: −Δ*S_m_^peak^* under 5 T at x = 5 is about 6.9% higher than the −Δ*S_m_^peak^* value at x = 4 and about 18.2% higher the −Δ*S_m_^peak^* value at x = 3. The increasing −Δ*S_m_^peak^* as well as *M_s_* with boron addition is most likely due to the exciting of free electrons in Fe atoms to a high spin state and thus strengthening the overall magnetic moments by adding of nonmagnetic B element. Therefore, the dependence of −Δ*S_m_^peak^* on *T_c_* of the Fe_92−x_Zr_8_B_x_ metallic glasses is contrary to the −Δ*S_m_^peak^*-*T_c_* relationship proposed by Belo et al. [44] in the RE-based glassy samples. The −Δ*S_m_^peak^* (under 5 T) vs. *T_c_* plots in several Fe-Zr-B-based amorphous samples (listed in Table 1), and their linear fitting (dash line) is illustrated in Figure 4b. −Δ*S_m_^peak^* for these Fe-Zr-B-based glassy ribbons increases monotonically with the Cutie temperature, which is possibly because the magnetic interactions in the Fe-Zr-B-based glass is not so complicated as the situation in the RE-based amorphous samples.

It is worthy to note that the Fe_87_Zr_8_B_5_ amorphous alloy exhibits a rather high −Δ*S_m_^peak^* at the temperature near 305 K. For instance, the −Δ*S_m_^peak^* under 5 T of the Fe_87_Zr_8_B_5_ glassy sample reaches to 3.25 J K^−1^ kg^−1^ at 305 K, which is comparable to that of the Fe_87_Zr_8_B_4_Sm_1_ amorphous ribbon (3.27 J K^−1^ kg^−1^ at 308 K) and the Fe_87_Zr_9_B_4_ amorphous ribbon (3.29 J K^−1^ kg^−1^ at 304 K), but is larger than that of the Fe_87_Zr_6_B_6_Cu_1_ (3 J K^−1^ kg^−1^ at 300 K) metallic glass [29,30,35]. In order to reveal the refrigeration efficiency of the Fe_87_Zr_8_B_5_ amorphous alloy, we calculate the temperature rise under an adiabatic condition (Δ*T_ad_*) of the sample according to

(1) $$\Delta T_{ad}\left(T,0\to H\right)=-\frac{T}{C_{p}\left(T\right)}\Delta S_{m}\left(T,0\to H\right)$$

**Figure 7:**
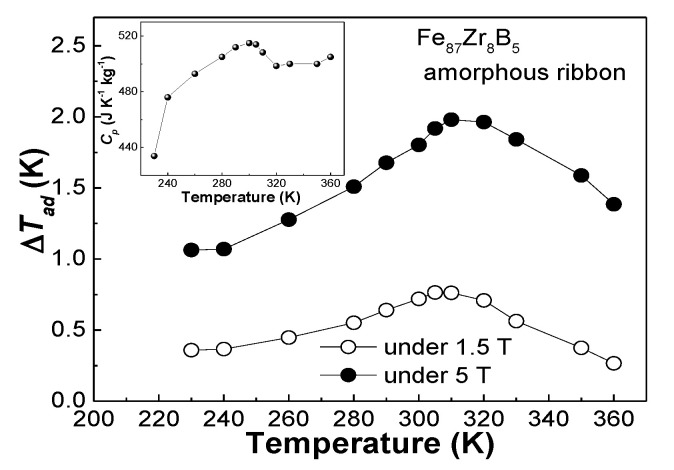
Δ*T_ad_*-*T* curve of the Fe_87_Zr_8_B_5_ amorphous alloy under the fields of 1.5 T and 5 T, the inset is the *C_p_*(*T*) curve of the amorphous alloy.

The Δ*T_ad_*-*T* curve of the Fe_87_Zr_8_B_5_ glassy ribbon is shown in Figure 7, the inset is the *C_p_*(*T*)) curve. The maximum Δ*T_ad_* for the Fe_87_Zr_8_B_5_ metallic ribbon is about 0.76 K under 1.5 T, and 1.98 K under 5 T, respectively.

## 4. Conclusions

In summary, the Fe_92−x_Zr_8_B_x_ (x = 3, 4, 5) glassy ribbons were successfully prepared. The magnetic and magnetocaloric behaviors of these glassy samples were studied. *T_c_* of the Fe_92−x_Zr_8_B_x_ glassy samples is about 271 K at x = 3, about 291 K at x = 4, and about 306 K at x = 5. It was found that *T_c_*, −Δ*S_m_^peak^*, and *M_s_* of the Fe_92−x_Zr_8_B_x_ amorphous samples show an increase trend with the boron content. The simultaneously increasing *M_s_*, *T_c_*, and −Δ*S_m_^peak^* with boron content in the Fe_92−x_Zr_8_B_x_ amorphous alloys is mostly likely attributed to the enhanced interaction between the Fe-B atoms by boron addition. The high −Δ*S_m_^peak^* and Δ*T_ad_* of the Fe_87_Zr_8_B_5_ metallic ribbon near 305 K indicate that the amorphous sample may be a good candidate for the magnetic refrigerants of a domestic magnetic refrigerator.

## Figures and Tables

**Table 1:** Curie temperature, *T_c_*, and −Δ*S_m_^peak^* of the several Fe-Zr-B-based amorphous samples.

Composition	−Δ*S_m_^peak^* * (J kg^−1^ K^−1^)					*T_c_* (K)	Ref.
	1 T	1.5 T	2 T	3 T	5 T		
Fe_89_Zr_8_B_3_	0.79	1.08	1.35	1.85	2.75	271	Present work
Fe_88_Zr_8_B_4_	0.88	1.20	1.50	2.06	3.04	291	
Fe_87_Zr_8_B_5_	0.94	1.29	1.61	2.19	3.25	306	
Fe_88_Zr_9_B_3_	0.94	1.28	1.59	2.16	3.17	286	[29]
Fe_87_Zr_9_B_4_	0.99	1.35	1.67	2.26	3.29	304	
Fe_86_Zr_9_B_5_	1.02	1.39	1.72	2.3	3.34	327	
Fe_88_Zr_8_B_4_	0.87	1.2	1.5	2.06	3.04	292	[34]
Fe_87_Co_1_Zr_8_B_4_	0.93	1.29	1.61	2.2	3.24	317	
Fe_86_Co_2_Zr_8_B_4_	0.98	1.35	1.69	2.31	3.38	340	
Fe_87_Zr_8_B_4_Sm_1_	0.98	1.33	1.65	2.24	3.27	308	[35]
Fe_86_Zr_8_B_4_Sm_2_	1.04	1.41	1.73	2.32	3.35	325	
Fe_85_Zr_8_B_4_Sm_3_	1.09	1.47	1.81	2.44	3.55	333	
Fe_87_Zr_7_B_4_Co_2_	1.01	1.38	1.72	2.34	3.42	333	[36]

* The maximum magnetic entropy change (−Δ*S_m_*) value in the −Δ*S_m_*-*T* curves.
